# Spontaneous Mesotheliomas in Germline *Bap1* Heterozygous Mice from Different Genetic Backgrounds

**DOI:** 10.3390/cancers17162692

**Published:** 2025-08-19

**Authors:** Yuwaraj Kadariya, Li Zhang, Eleonora Sementino, Eric Ross, Joseph R. Testa

**Affiliations:** 1 Cancer Prevention and Control Program, Fox Chase Cancer Center, Philadelphia, PA 19111, USA; yuwaraj.kadariya@fccc.edu (Y.K.);; 2 Biostatistics and Bioinformatics Facility, Fox Chase Cancer Center, Philadelphia, PA 19111, USA; li.zhang@fccc.edu (L.Z.);

**Keywords:** *BAP1* gene, mouse tumor models, mesothelioma, cancer predisposition, BAP1 tumor predisposition syndrome

## Abstract

**Background: ***BAP1* mutation carriers are predisposed to the development of mesothelioma. In mice, there is limited data and controversy about whether germline *Bap1* heterozygous mutations alone cause mesothelioma. However, a marked increase in mesothelioma incidence is observed in *Bap1*-mutant mice upon even minimal asbestos exposures. **Methods:** To address this issue, we investigated spontaneous mesothelioma development over the lifetime of a large cohort of *Bap1-*mutant and wild-type (WT) mice across several genetic backgrounds. To determine if the incidence of mesotheliomas in *Bap1*-mutant mice is significantly increased compared to WT mice, we performed statistical analyses using frequentist and Bayesian frameworks. In the Bayesian framework, to model the probability of disease occurrence, a non-informative prior was used for *Bap1*-mutant mice, whereas an informative prior for the WT group was derived from historical data spanning the animals’ lifetimes. Multiple strategies were employed to incorporate historical data and infer the informative prior, including a meta-analysis, assuming a consistent probability of mesothelioma across historical datasets, and applying Bayesian meta-analytic predictive priors derived from historical data. Posterior distribution was used, and a comparison was made using odds ratio, risk difference, and risk ratio. **Results:** Spontaneous mesotheliomas were detected in 2/329 *Bap1*-mutant and 0/227 WT mice from various genetic backgrounds. Using four statistical approaches, the results did not detect a significant difference in the probabilities of mesothelioma occurrence between *Bap1-*mutant and WT mice.  **Conclusions:** Based on these analyses, we cannot conclude that germline *Bap1*-mutant mice have a significantly increased risk of mesothelioma in the absence of asbestos exposure.

## Simple Summary

Individuals carrying a germline *BAP1* mutation are at increased risk of developing mesothelioma. In mice, there is limited information and controversy regarding whether germline *Bap1* heterozygous mutations alone cause mesothelioma, despite these mice being highly susceptible to even minimal asbestos exposure. To address this issue, we evaluated spontaneous mesothelioma development over the lifetime of a large cohort of *Bap1*-mutant mice and non-mutant, wild-type littermates across several genetic backgrounds. Spontaneous mesotheliomas were detected in 2/329 *Bap1*-mutant and 0/227 wild-type mice. Extensive analyses were performed using published (historical) lifetime data from wild-type mice and multiple statistical frameworks to determine whether mesothelioma incidence in *Bap1*-mutant mice differs from that of non-mutant mice. The analyses did not detect a significant difference in the probabilities of mesothelioma occurrence between *Bap1*-mutant and wild-type mice. Thus, we cannot conclude that germline *Bap1*-mutant mice have a significantly increased risk of mesothelioma in the absence of environmental exposure.

## 1. Introduction

The BAP1 tumor predisposition syndrome (BAP1-TPDS) is caused by heterozygous germline mutations in the BRCA1-associated protein-1 gene (*BAP1*). Carriers of a pathogenic *BAP1* mutation are at high risk for the development of benign *BAP1*-inactivated melanocytic tumors and various cancers, including mesothelioma, uveal and cutaneous melanomas, renal cell cancer, basal cell carcinoma, meningioma, and cholangiocarcinoma [1,2]. Germline *Bap1* heterozygous mice are at increased risk of developing mesothelioma upon asbestos exposure [3,4,5,6], even to minimal amounts [4,6], which is the leading environmental factor associated with the risk of this aggressive malignancy.

To determine if mice with germline *Bap1* heterozygous mutations are prone to the development of mesothelioma without exposure to asbestos fibers or other carcinogenic environmental agents, Kadariya et al. monitored spontaneous tumor development for up to 31 months in three genetically engineered mouse models (GEMMs) with different heterozygous inactivating *Bap1* mutations [5]. Each GEMM was in an FVB/N genetic background, including one with knockout of *Bap1* exons 6 and 7 (*Bap1^+/−^* mice). Two others had different knock-in mutations mimicking those found in two BAP1-TPDS families [7], one with a *Bap1* intron 6 splice site mutation resulting in a frameshift and premature stop codon, and the other with an exon 16 nonsense mutation. A similar spectrum of spontaneous tumors was identified in all three *Bap1*-mutant mouse models, with ovarian sex cord stromal tumors most frequently observed [5]. Spontaneous mesotheliomas were found in 2 of 93 *Bap1*-mutant mice and 0 of 43 wild-type (WT) littermates at publication [5].

Since then, we have followed the remaining FVB/N mice to the end of their lives. We also backcrossed our heterozygous *Bap1^+/−^* knockout mice to 129/Sv or C57BL/6 mice to assess spontaneous mesothelioma formation in these mouse strains. In addition, we investigated spontaneous mesothelioma development in a heterozygous *Bap1* mouse model with a different knockout mutation generated by another research group, also in the C57BL/6 background. We then used a rigorous set of Bayesian statistical analyses to interrogate the incidence of spontaneous mesotheliomas in *Bap1*-mutant versus WT mice in various genetic backgrounds. Based on these analyses, we report that there is insufficient evidence to support a statistically significant difference in the incidence of spontaneous mesotheliomas between germline *Bap1* heterozygous mice and WT mice in the absence of environmental carcinogenic exposure.

## 2. Methods

### 2.1. Generation of the Original Bap1-Knockout Mouse Model in FVB/N Background

Our *Bap1^+/−^* knockout GEMM has been reported previously [3,8]. Briefly, this mouse model was generated in an FVB/N genetic background using zinc finger nuclease (ZFN) technology. Custom ZFNs targeting *Bap1* were designed and validated in mammalian cells as described in detail elsewhere [8]. The *Bap1* knockout allele consisted of a deletion of exons 6 and 7, leading to a frameshift and predicted premature truncation of the Bap1 protein. The net effect is similar, but not identical, to that observed in a human BAP1-TPDS family having an intron 6 splice site mutation in *BAP1,* resulting in loss of exon 7 [7].

### 2.2. Generation of Bap1-Knockout Mice in 129/Sv and C57BL/6 Backgrounds

The original *Bap1* knockout mouse model in an FVB/N background was backcrossed separately to 129/Sv and C57BL/6 mice. The first-generation offspring were then backcrossed to either WT 129/Sv mice or WT C57BL/6 mice, each for nine generations, before expanding the colonies of *Bap1^+/−^* and *Bap1^+/+^* (WT) mice in a 129/Sv background and a C57BL/6 background.

### 2.3. Generation of Bap1 W and Bap1 L Knock-In Mouse Models in FVB/N Background

We also generated two GEMMs with inactivating *Bap1* mutations identical to those observed in the first two reported mesothelioma families with germline *BAP1* mutations, i.e., families W and L [7]. The *Bap1^+/W^* and *Bap1^+/L^* knock-in mice were generated in an FVB/N background using ZFN technology as previously described [5,8]. The *Bap1* L knock-in mutation generates the same stop codon observed in human family L [7]. The *Bap1* W knock-in mutation produces an mRNA that differs slightly from that seen in human family W due to sequence divergence in exon 7 between the human and mouse genomes; however, both mRNAs result in the loss of at least a portion of exon 7 and consequent premature truncation of the predicted gene product.

### 2.4. Constitutive Heterozygous Bap1- Knockout Mouse Model in a C57BL/6 Background

We also obtained *Bap1^+/−^* mice in a C57BL/6 background from Genentech, courtesy of Dr. Vishva Dixit. This GEMM was created by crossing mice possessing loxP sites flanking *Bap1* exons 4 and 5 with mice expressing Cre recombinase in most tissues throughout the body, effectively creating a “whole-body” knockout of the *Bap1* gene [9]. Compared with their WT littermates, these constitutive heterozygous *Bap1^+/−^* mice express about half the Bap1 protein in mesothelial cells [4].

At least 50 mice with each genotype per genetic background were followed throughout their lifetime for mesothelioma development. All mouse studies were conducted in accordance with the NIH’s Guide for the Care and Use of Laboratory Animals and a protocol approved by our Institutional Animal Care and Use Committee (IACUC).

### 2.5. Analysis of Spontaneous Tumors in Bap1-Mutant Mice

All mice were monitored daily, including weekends, and sacrificed upon evidence of labored breathing, weight loss of >10% body weight, lethargic behavior, poor grooming, abdominal bloating, hunched back, and/or mobility limitations, or when tumor burden was evident, following a protocol approved by Fox Chase Cancer Center’s IACUC. Mice were sacrificed by CO_2_ asphyxiation followed by cervical dislocation to make sure that animals would not resuscitate, per IACUC guidelines. At the time of euthanization, all organs in the thoracic, abdominal, and pelvic cavities were carefully examined for evidence of tumor lesions or overt malignancy. Tissues were collected, fixed in 10% formal saline fixative (Mercedes Scientific, Lakewood Ranch, FL, USA) overnight, and sent for histopathological analysis. Formalin-fixed, paraffin-embedded (FFPE) samples were cut into 5-μm sections and mounted onto positively charged microscope slides. Sections were dewaxed in xylene and hydrated through a graded ethanol series before staining with hematoxylin and eosin (H&E) and histopathological analysis; each of thesel reagents was from Thermo Fisher Scientific, Waltham, MA, USA. Heat-induced antigen retrieval was performed, followed by blocking of endogenous peroxidase activity. All H&E and immunohistochemical (IHC) staining was performed by our Histopathology Facility at Fox Chase Cancer Center (Philadelphia, PA, USA), and all histopathologic assessments were independently performed by two experienced experimental animal pathologists as described [5]. Markers used to confirm the diagnosis of mesothelioma included mesothelin antibody D-16 (sc-27702) and WT1 antibody C-19 (sc-192), each from Santa Cruz Biotechnology (Dallas, TX, USA), and pan-cytokeratin antibody Z0622 from Dako (Glostrup, Denmark). Bap1 expression was determined using antibody A302-242A from Bethyl Laboratories (Montgomery, TX, USA). Murine samples previously shown to express high levels of each protein investigated were used as positive controls. As a negative control, the primary antibody was replaced with normal mouse or rabbit IgG to confirm the absence of specific staining.

### 2.6. Genotyping

Tail DNA samples were obtained through digestion and purification with a Gentra DNA Extraction Kit (Qiagen, Valencia, CA, USA). The genotyping of the original *Bap1*-knockout mouse model in the FVB/N background has been described elsewhere [3,8]. The same primers and genotyping conditions were used for the *Bap1*-knockout mice in 129/Sv or C57BL/6 backgrounds. For *Bap1^+/W^* and *Bap1^+/L^* knock-in mice, the genotyping has been described elsewhere [5]. For the *Bap1* knockout mouse model from Genentech, we used the same primers used in their report [9].

### 2.7. Identification of Published Datasets

We considered literature databases that use WT mice to screen for mesothelioma without exposure to asbestos or other exogenous factors. Studies were included regardless of mesothelioma location (pleural, pericardial, peritoneal, or testicular) and tumor subtype (epithelioid, sarcomatoid, or mixed). One key consideration is that mesothelioma may develop in the later stages of a mouse’s lifespan. Therefore, we focused on studies with a follow-up duration that spanned the mouse’s lifetime or at least up to about 2.5 years.

### 2.8. Statistics

#### 2.8.1. Fisher’s Exact Test

To compare the incidence of mesothelioma between WT mice and germline *Bap1* heterozygous mice, we first performed Fisher’s exact test. This test is particularly appropriate for small sample sizes and scenarios with low expected frequencies.

#### 2.8.2. Bayesian Hierarchical Model

To incorporate historical data and provide a more robust analysis, we also employed a Bayesian framework. Let $P_{w}$ and $P_{m}$ denote the probabilities of the occurrence of spontaneous mesothelioma among WT mice and germline *Bap1* heterozygous mice, respectively. Our analysis used a Bayesian hierarchical model [10] to assess the probability of spontaneous mesotheliomas in the WT group. The observed counts of mesothelioma occurrences in each group, denoted $Y_{w}$ and $Y_{m}$, were modeled using the following binomial distributions.

(1) $$Y_{w}\sim Binomial(n_{w},P_{w})$$

(2) $$Y_{m}\sim Binomial(n_{m},P_{m})$$

(3) $$P_{m}\sim Beta(1,1)$$

(4) $$P_{w}\sim Beta(\alpha ,\beta )$$

Here, $n_{w}$ and $n_{m}$ represent the total number of mice in the WT and *Bap1*-mutant groups, respectively.

Using the Bayesian framework [10], inference is made by combining information from newly observed data with a prior distribution, representing prior knowledge about the parameters before observing new data. Translational animal experiments are often conducted with small sample sizes in each experimental group to uphold animal welfare standards [11]. In such cases, the choice of prior distribution plays a crucial role in posterior inference. A well-specified prior that accurately reflects existing knowledge can help stabilize posterior estimates, improve precision, and reduce the influence of extreme observations. Historical data from previously conducted studies that have investigated the same outcome in similar animal models can be used to derive prior distributions for the analysis of the new experiment.

For a binomial distribution, we considered its conjugate prior, the beta distribution. We did not have historical data for the mutant group, so we used a non-informative prior Beta(1,1), which assumes a uniform probability density, reflecting minimal prior knowledge. In contrast, we applied an informative prior using a Beta(α,β) distribution based on historical data for the WT group. In this case, the historical data reflect the estimated probability of mesothelioma, based on WT data, before observing the new data. This information may be used to determine the α,β parameters of the informative prior for the current WT data.

To incorporate historical data and infer the α,β parameters, we considered three different strategies: (1) assuming the same probability of mesothelioma across the historical data and using its estimated posterior distribution; (2) performing a meta-analysis to combine the mesothelioma occurrence rate from historical data and using the combined rate information; and (3) applying Bayesian meta-analytic predictive (MAP) priors derived from historical data. To incorporate historical data as appropriate prior distributions in (1), the non-parametric estimates of the posterior distribution, represented by Markov chain Monte Carlo (MCMC) draws, were approximated using parametric distributions [12].

*Same mesothelioma occurrence rate across the historical WT data:* To implement this, we assume a hierarchical model where the probabilities of mesothelioma across all historical data are the same, denoted as $P_{historical}$. Under this model, the number of occurrences of mesothelioma within a study *i*, $x_{i}$ is as follows.

(5) $$x_{i}\sim Binomial(n_{i},P_{historical})$$

(6) $$P_{historical}\sim Beta(1,1)$$

Here, $P_{historical}$ is assigned a non-informative prior. To account for uncertainty across studies, we incorporate the 95% credible interval of $P_{historical}$ from the posterior distribution. The resulting point estimate and credible interval are then used to derive the α,β for $P_{w}$.

*Meta-analysis to combine mesothelioma occurrence rate in historical WT data:* Here, we assume that the probability of occurrences of mesothelioma within a study *i* is $p_{i}$, allowing the probability to vary across studies. To estimate the overall effect across multiple studies, we employed an inverse variance weighting approach combined with a logistic transformation to estimate the overall effect across various studies. The inverse variance weighting method assigns greater weight to studies with lower variance (i.e., larger sample sizes), allowing more precise studies to have a greater influence on the overall effect estimate [13]. To address the issue of studies reporting zero occurrences, we applied a continuity correction. The methodology follows the inverse variance approach described in Borenstein et al. [13]. The pooled estimate of the common probability effect and its associated uncertainty are estimated as follows [14].

(7) $$\theta_{i}=log(\frac{p_{i}}{{1-p}_{i}})$$

(8) $$p_{i}=\frac{x_{i}+0.5}{n_{i}+1}$$

(9) $$\hat{\theta }=\sum \frac{w_{i}\vartheta_{i}}{w_{i}},$$

(10) $$w_{i}=\frac{1}{{SE(\theta_{i})}^{2}+\tau^{2}}$$

Here, $x_{i}$ and $n_{i}$ refer to the number of events and the total sample size in study *i*. Continuity correction (0.5) is applied when $x_{i}=0.$ Between-studies variance $\tau^{2}$ is estimated using the DerSimonian and Laird method [15].

*Meta-analytic predictive priors for historical WT data:* We also considered the MAP approach [16], a Bayesian method that directly constructs an informative prior distribution based on historical data. Unlike traditional meta-analysis, where the primary outcome is Equation (9), the MAP approach focuses on predicting $\theta^{*}$ in the new study. The goal is to derive a predictive prior that captures relevant information from historical data while accounting for additional uncertainty when extrapolating to a new study. Here, $x_{i}$ and $n_{i}$ are the number of events and the total sample size in study *i*, and the mixture prior distribution for Equation (11) in a new study is estimated as follows:

(11) $$p^{*}=\frac{exp(\theta^{*})}{1+exp(\theta^{*})}$$

(12) $$x_{i}\sim Binomial(n_{i},p_{i})$$

(13) $$\theta_{i}=log(\frac{p_{i}}{{1-p}_{i}})=\beta_{0}+\mu_{i}$$

(14) $$\mu_{i}\sim N(0,\tau^{2})$$

(15) $$p^{*}\sim \sum_{k=1}^{K}w_{k}Beta(p^{*}|a_{k},b_{k})$$

The weights and the hyperparameter k can be obtained as maximum-likelihood (ML) estimates in Kullback–Leibler divergence [17].

The posterior distributions of $P_{w}$ and $P_{m}$ were estimated using the proposed model. In MCMC methods, the posterior of the parameter distribution is approximated by a series of MCMC draws. To compare $P_{w}$ and $P_{m},\;$ we proposed the use of multiple criteria, including the odds ratio (OR), risk ratio (RR), and risk difference (RD). If the occurrence rates of mesothelioma are the same in both groups, then OR and RR equal 1, while RD equals 0. If the occurrence of mesothelioma is higher in the *Bap1*-mutant group, then OR and RR will be greater than 1, and RD will be greater than 0. We formally tested the following null and alternative hypotheses as shown below.

(16) $$H_{0}:\;\mathrm{OR}=1\;\mathrm{vs}.\;H_{A}:\;\mathrm{OR}\;\neq 1$$

(17) $$H_{0}:\;\mathrm{RR}=1\;\mathrm{vs}.\;H_{A}:\;\mathrm{RR}\;\neq 1$$

(18) $$H_{0}:\;\mathrm{RD}=0\;\mathrm{vs}.\;H_{A}:\;\mathrm{RD}\;\neq 0$$

Statistical significance in a frequentist context can be interpreted within a Bayesian framework using Bayesian credible intervals [10]. The posterior quantiles generated from the MCMC samples can be used to evaluate these hypotheses using a 95% equal-tailed credible interval (95% probability that the parameter lies within this interval). Specifically, for OR and RR, if the 95% credible interval does not include 1, and for RD, if the 95% credible interval does not include 0, we reject the null hypothesis and conclude that the rate of mesothelioma in germline *Bap1*-mutant mice is greater than in WT mice.

Under a Bayesian approach, it is possible to calculate the posterior probability that the OR is greater than a given value. Since mesothelioma is a rare event, occurring infrequently in both the WT and *Bap1-*mutant groups, the OR closely approximates the relative risk. However, due to the low event rates, both OR and RR may be highly skewed. To address this, we also consider alternative cutoffs, such as 2 and 5, to assess the strength of the association.

All statistical analyses were performed using R software (version 4.0.5). The Bayesian hierarchical method was implemented with the RStan package [18] (version 2.32.6). The MCMC settings were defined with four chains running for 4000 iterations, setting the warmup as half of the total iterations, a thinning rate of 1, and random initial values for the sampler. Meta-analysis was performed using the meta package (version 8.0-2). MAP was performed using the RBesT package (version 1.8-1).

## 3. Results

### 3.1. Spontaneous Mesotheliomas in Bap1-Mutant Mice and WT Littermates

In our earlier study of spontaneous tumors in three germline *Bap1*-mutant heterozygous GEMMs (*Bap1^+/−^*; *Bap1^+/W^*; *Bap1^+/L^*), each in an FVB/N background, animals were followed up to 31 months of age [5]. Two mesotheliomas (one in a *Bap1^+/−^* mouse; the second in a *Bap1^+/W^* mouse) were found in 93 mice from these three *Bap1*-mutant mouse models. No mesotheliomas were found in 43 WT mice, although the difference in mesothelioma incidence between *Bap1*-mutant and WT groups was not statistically significant (*p* > 0.05, Fisher’s exact test) [5].

In the current study, we report that we continued to monitor the remaining mice from these three FVB/N cohorts until their end of life, up to about 35 months in some animals. We also followed 54 additional *Bap1^+/−^* FVB/N mice and 20 WT littermates, specifically seeking to expand our dataset to identify any further evidence of mesothelioma formation over the entire lifetime of animals in this genetic background. No new spontaneous mesotheliomas were seen. Similarly, cohorts of *Bap1^+/−^* mice and WT littermates in 129/Sv or C57BL/6 backgrounds were followed until the end of life, and no spontaneous mesotheliomas were observed. Likewise, no spontaneous mesotheliomas were identified in Genentech’s *Bap1^+/−^* or *Bap1^+/+^* (WT) mice in a C57BL/6 background. Overall, in our data from all genetic backgrounds (FVB/N, 129/Sv, C57BL/6), 2 of 329 mice with a germline *Bap1* heterozygous mutation developed mesothelioma, compared to 0 of 227 WT mice. Using the exact binomial test, the observed rates of mesothelioma and their 95% confidence intervals in the *Bap1*-mutant and WT groups were 0.006 [0.0007, 0.022] and 0 [0.00, 0.016], respectively. To statistically assess whether the proportion of mesothelioma occurrences differs between the *Bap1*-mutant and WT groups, we first conducted a Fisher’s exact test, which is particularly suitable for small sample sizes and instances where expected frequencies are low. The resulting *p*-value from Fisher’s exact test is 0.516, indicating no statistically significant difference in the occurrence of mesothelioma between the two groups. The incidence of mesotheliomas in the six germline *Bap1* heterozygous mouse models and their WT littermates is presented in Table 1.

### 3.2. Historical Data Identified

Based on the study by Nielsen et al. [19], six sources were identified that contained data on background mesothelioma rates in WT mice that were neither exposed to exogenous toxicants nor genetically modified. Most of the mice from these historical sources had limited follow-up times of 78 to 104 weeks. Based on studies with follow-up times closer to the end of life or nearly so (128−135 weeks), two studies from these sources were selected for our historical control set (Table 2). These studies were used to establish prior information about the probability of mesothelioma rates in WT mice, $P_{w}$ [20,21].

### 3.3. Results Using the Same Mesothelioma Occurrence Rate Across the Historical Data

**Figure 1:**
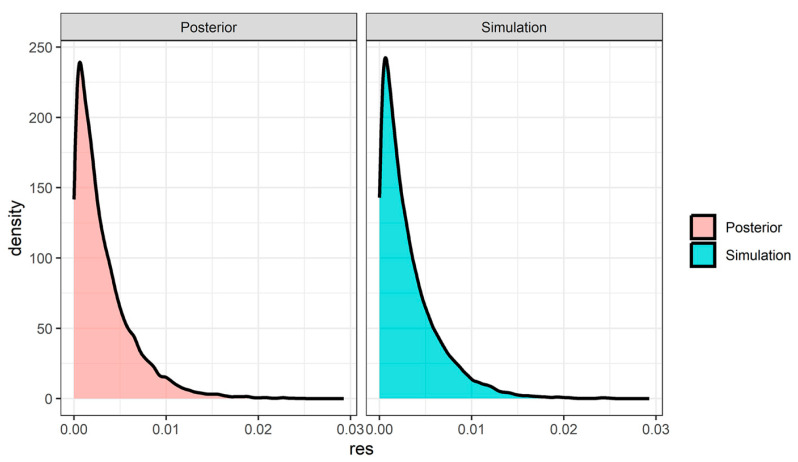
Posterior distribution of $P_{historical}$ from Markov chain Monte Carlo (MCMC) draws (***left** panel*) and the density distribution of a simulation from Beta (1,308) (***right** panel*). Note that the two distributions are well matched.

Using the historical data, we estimated the posterior mean and 95% credible interval for $P_{historical}$ as 0.00324 [0.00008, 0.0119]. Based on this information, we derived the estimated values of α and β to ensure that the resulting beta distribution appropriately captures the posterior information. The estimated values for α and β were 1 and 308, respectively. Figure 1 shows the posterior distribution of $P_{historical}$ from MCMC (*left panel*) and the density distribution of a simulation from Beta (1,308) (*right panel*). The two distributions match perfectly.

**Figure 2:**
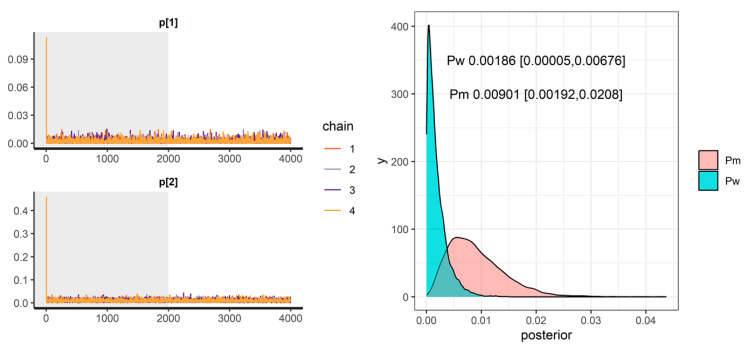
Trace plot (***left** panel*) and posterior distribution (***right***) of $P_{m}$ and $P_{w}$. The values represent their posterior means and 95% credible interval.

We next applied Bayesian analysis with a non-informative prior for germline *Bap1* heterozygous mice and an informative Beta (1,308) prior for the WT group. The MCMC was run, and convergence was assessed using R-hat statistics and trace plots (Figure 2, *left panel*), confirming no convergence issues. Therefore, we confidently summarized the posterior distributions of the parameters. Figure 2 (*right panel*) provides the posterior distribution of $P_{w}$ and $P_{m}$. As shown, the posterior probability distribution for $P_{w}$ is narrower than that of $P_{m}$, reflecting the influence of prior information from the historical data. The 95% credible interval for $P_{w}$ ranges from 0.00005 to 0.00676, whereas for $P_{m}$, it ranges from 0.00192 to 0.02079. Notably, the 95% credible intervals for $P_{w}$ and $P_{m}$ do overlap.

**Figure 3:**
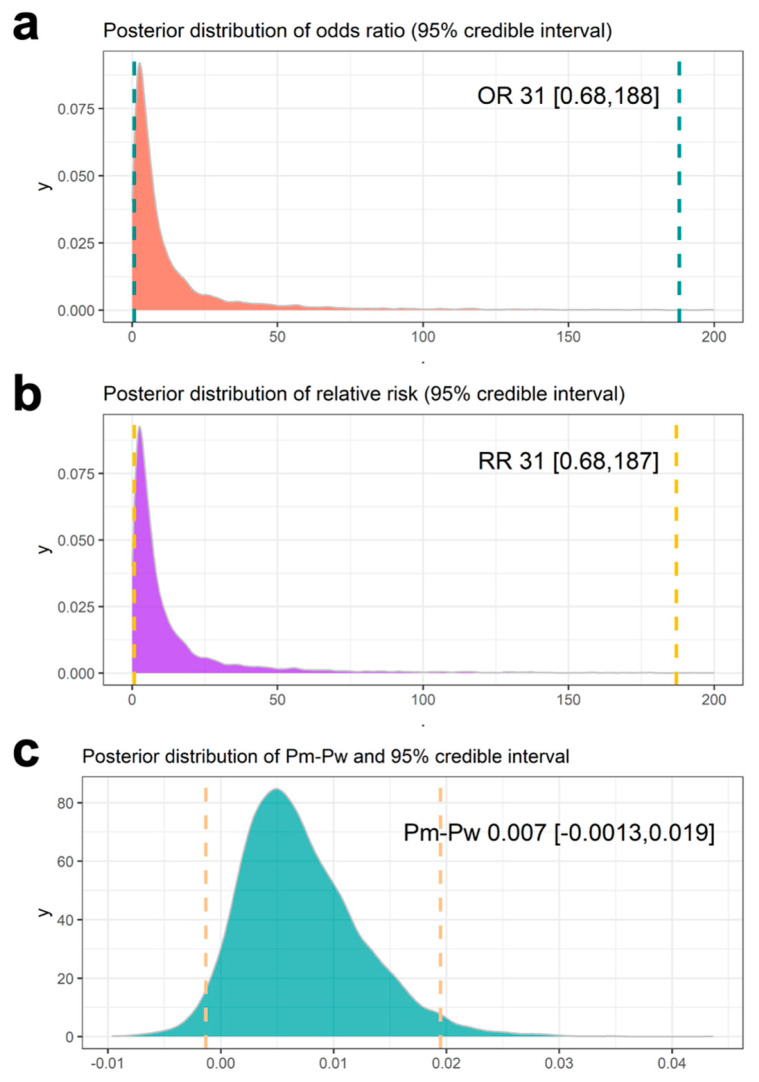
Posterior distribution of odds ratio (**a**), relative risk (**b**), and $P_{m}$ and $P_{w}$ (**c**). The values represent their posterior mean and 95% credible interval. The dotted lines indicate the 95% credible intervals.

We then examined the posterior distributions of the OR, RR, and RD to compare the occurrence of mesothelioma between *Bap1-*mutant and WT mice. As expected, the posterior distributions of OR and RR were highly skewed to the right (Figure 3a and Figure 3b, respectively). The 95% credible interval for OR ranged from 0.68 to 188, with a similar range observed for RR. This large range reflects the rarity of mesothelioma in both mouse groups, leading to substantial uncertainty in the estimated ratios. The estimated mean RD between $P_{m}$ and $P_{w}$ was 0.007, with a 95% credible interval ranging from −0.00134 to 0.01946 (Figure 3c). The lower bound of the credible intervals for OR and RR was less than 1, and for RD, it was clearly below 0. Based on these 95% credible intervals, there is no statistically significant difference between $P_{m}$ and $P_{w}$, indicating that we cannot conclude that the occurrence of mesothelioma is higher in germline *Bap1* heterozygous mice than in WT mice after incorporating the historical data.

As an additional secondary analysis, we further assessed the probability of observing at least two mesothelioma cases under the null hypothesis that $P_{m}=P_{w}$. We performed 100,000 simulations, each generating a dataset of 329 mice (matching the sample size of the *Bap1*-mutant group) under the assumption that $P_{w}=0.00186$, the posterior mean estimate of $P_{w}$. For each simulated dataset, we recorded whether two or more mesothelioma cases were observed. The proportion of times this occurred was computed across the simulation scenarios. Based on these simulations, the probability of observing two or more mesothelioma cases under the assumption that $P_{m}=P_{w}$ was 13%. This suggests that the observed data are not highly unusual under the null hypothesis, further supporting the conclusion that there is no strong evidence for an increased mesothelioma incidence in *Bap1-*mutant compared to WT mice. Furthermore, using the posterior samples from our Bayesian analysis, we estimated that the probability of the OR exceeding 2 is 83%, while the probability of the OR exceeding 5 is 57%. These probabilities provide evidence suggesting an increased risk of mesothelioma in *Bap1-*mutant mice compared to WT mice. However, given the wide credible intervals and the 95% credible interval, we cannot conclude that a germline *Bap1* heterozygous mutation in mice is associated with an increased incidence of mesothelioma.

### 3.4. Results Using Meta-Analysis to Combine Mesothelioma Occurrence Rate in Historical Data

**Figure 4:**
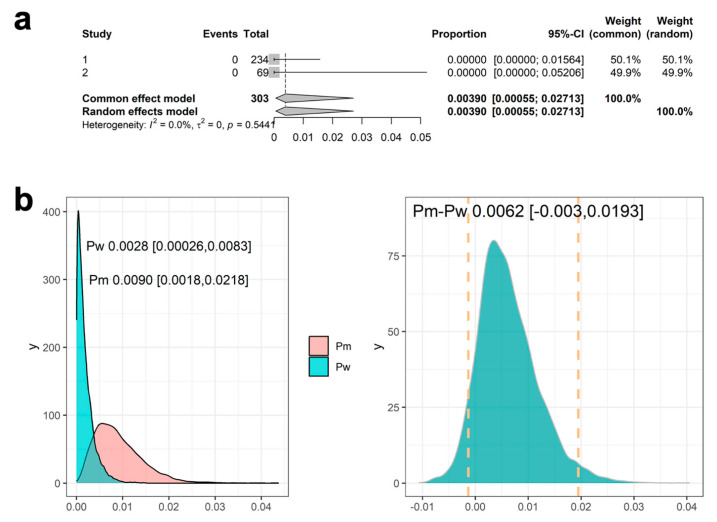
Estimates (point and 95% CI) using meta-analysis (**a**), and posterior distribution of $P_{w}$ and $P_{m}$ and risk difference (RD) between $P_{m}$ and $P_{w}$ using prior derived from meta-analysis (**b**). The dotted lines in panel (**b**) (right panel) indicate the 95% credible intervals.

Using the inverse variance weighting approach combined with a logistic transformation, the pooled results from the meta-analysis estimate the proportion of mesothelioma from the historical data to be 0.0039 (Figure 4a) with a 95% confidence interval (CI) of [0.00055, 0.02713]. The α and β parameters derived to ensure that the resulting beta distribution appropriately captures the 95% CI are 0.74 and 113.57, respectively. With this informative prior for $P_{w}$, the posterior distribution of $P_{w}$ and $P_{m}$, along with the RD between $P_{m}$ and $P_{w},$ are shown in Figure 4b. The mean RD was 0.0068, with a 95% credible interval ranging from −0.003 to 0.0198. Similarly, the lower bound of the credible intervals for RD was below 0. Based on these 95% credible intervals, we did not observe a statistically significant difference between $P_{m}$ and $P_{w}$.

### 3.5. Results Using Meta-Analytic Predictive Priors for Historical Data

**Figure 5:**
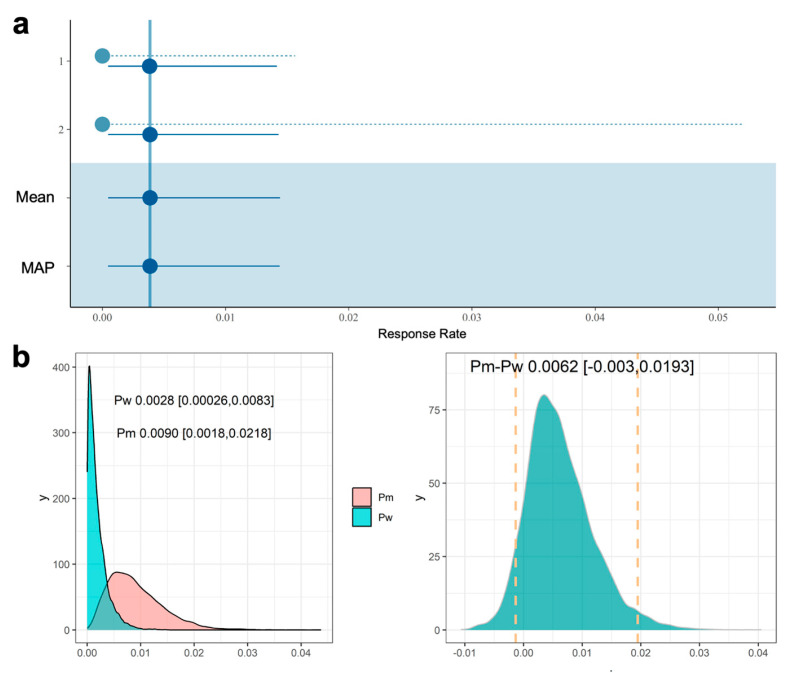
Predictions (estimates, 95% intervals) derived from meta-analytic-predictive (MAP) approaches (**a**), and posterior distribution of $P_{w}$ and $P_{m}$ and RD between $P_{m}$ and $P_{w}$ using prior derived from MAP (**b**). The dotted lines in panel (**b**) indicate the 95% credible intervals.

Given the low heterogeneity between the two studies, we applied a half-normal prior for τ with parameters (0,1/16), and for $\beta_{0}$, we assumed a normal distribution with mean 0 and variance 4. Figure 5a presents the predictions derived using the MAP approach, based on the observed log-odds for each trial, and assuming normally distributed random effects on the log-odds scale. The MAP sample yielded a posterior mean of 0.0047, with a corresponding 95% credible interval of [0.00046, 0.0144].

The mixture k providing the best Akaike Information Criterion (AIC) value was selected as 1. The derived α and β parameters were 1.70 and 357.01, respectively. The posterior distribution of $P_{w}$ and $P_{m}$, along with the RD between $P_{m}$ and $P_{w}$, are shown in Figure 5b. The mean RD was 0.0062, with a 95% credible interval ranging from −0.003 to 0.019. This result is very similar to the one using meta-analysis.

Using the same mesothelioma rate across the historical data, we obtained a point estimate of $P_{historical}$ 0.00324 with a 95% credible interval of [0.00008, 0.0119] for the probability of mesothelioma in WT mice. This estimate was compared to the meta-analysis estimate of 0.0039 with a 95% confidence interval of [0.00055, 0.02713] and the MAP estimate of 0.0047 with a 95% credible interval of [0.00046, 0.0144]. The first method provides the most conservative estimate, yielding both a lower point estimate and narrower credible interval bounds. Despite using the most conservative prior, we still did not detect a significant difference between the probabilities of mesothelioma occurrence in *Bap1*-mutant ($P_{m}$) and WT ($P_{w}$) mice. The consistent results using four statistical approaches (Fisher’s exact test and all three methods in the Bayesian framework comparing $P_{w}$ and $P_{m})\;$ indicate that, given the available data, there is insufficient evidence to support a statistically significant difference in the incidence of spontaneous mesotheliomas between germline *Bap1* heterozygous mice and WT mice.

## 4. Discussion

In 2016, we reported spontaneous mesotheliomas in 2 of 93 FVB/N mice with different germline *Bap1* heterozygous mutations compared to 0 of 43 WT littermates that had been sacrificed at the time of publication [5]. Based on those findings, some investigators have concluded that germline mutations of certain cancer predisposition genes, such as *Bap1*, are powerful and may cause mesothelioma without environmental asbestos exposure [22]. Since our original report, however, no additional mesotheliomas have been identified in 54 additional *Bap1^+/−^* FVB/N mice followed to their end of life. Similarly, no spontaneous mesotheliomas were observed in heterozygous *Bap1^+/−^* mice in either 129/Sv or C57BL/6 backgrounds. In addition, no spontaneous mesotheliomas were seen in germline *Bap1^+/−^* mice in a C57BL/6 background obtained from Genentech. These new mouse data, in several different genetic backgrounds, do not support the conclusion that germline *Bap1* heterozygous mice have an increased risk of mesothelioma compared to their WT littermates.

Recently, Nielsen and colleagues [19] performed a Bayesian analysis to reexamine the data from our 2016 report [5] to determine if the rate of spontaneous mesotheliomas among germline *Bap1* heterozygous mice is greater than among WT mice. Using these base data independently with a non-informative prior in the Bayesian framework, they determined that the posterior probability of a difference in these rates was not statistically significant (probability of OR being > 1 = 68.7%), similar to what we had concluded in our report using Fisher’s exact test [5]. The investigators then created a historical control dataset (HCD) of 8627 WT mice, consisting of several strains not exposed to asbestos, of which 8 developed spontaneous mesotheliomas [19]. By reapplying their Bayesian evaluation of our base data with their HCD, the posterior probability of a difference in the rates of mesothelioma occurrence between *Bap1-* mutant mice and WT mice increased to >99.9%. Several additional Bayesian approaches for incorporating historical data while controlling for heterogeneity between datasets led the investigators to conclude that the incidence of spontaneous mesotheliomas in *Bap1*-mutant mice is significantly higher than that of WT mice (96.7–99.5% probability that the OR is >1 and 93.2–97.9% probability that it is >2).

There are several limitations in the assessment by Nielsen et al. [19], including the fact that the WT mice in their HCD were not housed at the exact location or under the same conditions as the animals used for comparison, i.e., the *Bap1*-mutant mice from the report by Kadariya et al. [5]. As reviewed by Everitt [23], the incidence of spontaneous tumors can be influenced by many factors, including genetic background, diet, housing conditions, infection, hormones, age, etc. In addition, all mice used in our report [5] were in an FVB/N background, whereas only 7% of the mice in Nielsen and colleagues’ HCD were FVB/N mice, with the remaining 93% being a different strain (CD-1 mice). This is noteworthy because different mouse strains can have significantly different rates and types of spontaneous tumors (reviewed in [24]). Furthermore, in one of the studies included in the HCD used by Nielsen et al. [19], from an extensive report by Giknis and Clifford [25], more than 2716 CD-1 mice were followed for only 78 weeks. No mesotheliomas were observed then, whereas 6 of 3,520 CD-1 mice monitored for up to 104 weeks developed spontaneous mesotheliomas. In another report in the HCD used by Nielsen and colleagues [19], Maita et al. [26] identified mesotheliomas in 2 of 1,781 CD-1 mice used in control groups from eleven 2-year carcinogenicity studies. This research group also reported that the average mortality of male and female CD-1 mice at about 2 years of age was 66.4% and 63.3%, respectively, indicating that about 35% of their CD-1 mice were not followed until the end of life to determine if they might have developed additional spontaneous tumors, including mesotheliomas. Since spontaneous mesotheliomas appear to occur later in life in CD-1 mice [25], the reported incidence of these tumors in the HCD used by Nielsen et al. [19] is likely to be underestimated.

As mentioned earlier, spontaneous mesotheliomas were observed in two *Bap1*-mutant mice in an FVB/N background but were not seen in any *Bap1*-mutant mice in C57BL/6 or Sv129 backgrounds. Such differences between strains could mean that FVB/N mice have other genetic (or epigenetic) factors that cooperate with the *Bap1* mutation to make them more susceptible to the development of mesothelioma than those in the other two mouse strains. Alternatively, mesothelial cells of C57BL/6 and 129/Sv mice may differentially express “protective” genes compared to FVB/N mice, resulting in decreased sensitivity to the deleterious effects of a *Bap1* mutation. In the absence of a *Bap1* mutation, wild-type CD-1 mice develop occasional spontaneous mesotheliomas, whereas WT mice from other strains reported here do not. Such discrepancies may be due to differences in the expression of pro- or anti-tumorigenic genes between different mouse strains.

Nielsen et al.’s report included only one study of *Bap1*-mutant mice: our original report of 93 animals [5]. To expand the number of *Bap1*-mutant mice, we conducted additional studies on *Bap1*-mice in various genetic backgrounds. We monitored 236 new germline *Bap1* heterozygous mice, in several different backgrounds, over their lifetime for the development of spontaneous mesothelioma. When these new data were evaluated using rigorous Bayesian analytical methods and an HCD consisting of mice also followed to the end, or nearly the end, of life, our findings contradict those of Nielsen et al. [19]. Our analysis of the expanded set of mice from several different genetic backgrounds demonstrates that germline *Bap1* heterozygous mice do not have a statistically significantly higher incidence of spontaneous mesotheliomas compared to WT mice. Although germline *BAP1* heterozygous mutations are known to *predispose* to human mesothelioma [7], the findings presented here indicate that germline *Bap1* mutations do not result in a statistically significant risk of mesothelioma in mice. However, in vivo studies have documented that germline *Bap1* heterozygous mice are highly susceptible to mesothelioma upon exposure to even minimal amounts of asbestos fibers [4,6]. These investigations have demonstrated that exposures to very low doses of crocidolite (total 0.1–0.5 mg, compared to 3–5 mg generally used in such experiments) induced mesotheliomas in 36–47% of heterozygous *Bap1*-mutant mice versus about 10% of similarly exposed WT littermates. Notably, these *Bap1^+/−^* mice developed mesothelioma at a rate comparable to WT mice that were exposed to 8- to 10-fold higher doses of asbestos [4,6]. No mesotheliomas were observed in control unexposed *Bap1*-mutant or WT mice. These findings support a gene–environment interaction associated with the high incidence of mesothelioma observed in heterozygous *Bap1*-mutant mice exposed to even trace amounts of asbestos.

The interaction of genetic predisposition and asbestos, especially low exposures, has also been established in humans [27,28]. Dianzani and colleagues reviewed cumulative asbestos exposures from a series of their studies on malignant pleural mesothelioma (MPM) patients; their 23 MPM patients carrying a germline pathogenic variant (PV) in a cancer-predisposing gene showed a significantly lower cumulative asbestos exposure compared with the 189 MPM patients without germline PVs (*p* < 0.0001) [28]. The mean quantitative asbestos exposure among both controls and patients with germline PVs was 2.7 f/mL-y compared to 21.7 f/mL-y among patients with MPM that did not have germline mutations in *BAP1* or other cancer-predisposing genes [28]; thus, the mean quantitative asbestos exposure in MPM patients without germline PVs was 8.0-fold higher than in those with germline PVs. Moreover, the fact that similar levels of asbestos exposure were observed in patients with germline PVs and controls from the same geographic area further supports the crucial role of gene-environment interactions in asbestos-related carcinogenesis [28].

Other investigators have also reported that mesothelioma patients with germline PVs generally have minimal known asbestos exposures [29,30]. In these two studies, 13 of 18 (72%) patients with germline *BAP1* mutations reported definite or possible asbestos exposure. However, asbestos exposure was self-reported and not based on independent quantitative measurements, a study limitation noted in one of the reports [30].

The fact that most BAP1-TPDS-related neoplasms arise in middle-aged and older adults, and that the penetrance for any cancer type associated with this syndrome is less than 100% (e.g., 25–35% for mesothelioma [2,22,30] suggests that genomic alterations in addition to *BAP1* loss are required for cancer formation [31]. For instance, in *BAP1*-related uveal melanoma, mutations in genes encoding components of the Gq signal transduction pathway are also needed to cause the disease [31,32]. BAP1 significantly regulates DNA repair by homologous recombination and cell death in some cell types [31]. However, in uveal melanoma, *BAP1*-mutant cells do not exhibit strong evidence of DNA damage repair defects [33]. In the mouse, in addition to a germline *Bap1* mutation, other factors may also be required to cause mesothelioma, such as mutations in genes encoding components of a currently unknown cell signaling pathway, environmental factors such as carcinogenic mineral fibers and toxins, radiation, or simply the accumulation of random mutations due to the aging process. Some of these same factors may also contribute to the rare mesotheliomas observed in WT mice of certain strains, such as CD-1 [25,26].

The incidence rate of mesotheliomas in human *BAP1*-mutation carriers is much higher than the rate of spontaneous mesotheliomas in mice carrying heterozygous *Bap1* germline mutations. Besides the fact that GEMMs do not necessarily recapitulate their human disease counterpart, there are critical inherent differences that may contribute to this discrepancy. Studies by several groups have documented that most mesothelioma patients carrying a germline mutation of *BAP1* or other cancer-predisposing genes have been exposed to asbestos. However, the exposure amounts are often lower in these individuals than in mesothelioma patients without germline mutations [28,29,30]. In contrast, *Bap1*-mutant mice employed for studies of spontaneous tumors are housed in a “protected” environment without exposure to asbestos. Yet, when they are exposed to even trace amounts of asbestos fibers, *Bap1*-mutant mice develop a high incidence of mesothelioma [4,6]. Therefore, *Bap1*-mutant mice may serve as ideal experimental models for testing the tumorigenic effects of a germline *Bap1* mutation on exposures to an environmental carcinogen or for preclinical studies of chemopreventive agents.

## 5. Conclusions

Using multiple statistical approaches, our results did not detect a significant difference between the probabilities of mesothelioma occurrence in *Bap1-*mutant and WT mice. Thus, we cannot conclude that germline *Bap1* heterozygous mice have an increased risk of spontaneous mesothelioma compared to WT mice. However, given that even trace amounts of asbestos induce a high incidence of mesothelioma in *Bap1*-mutant mice compared to WT mice [4,6], this suggests that germline *Bap1* mutations create a highly susceptible setting for a gene-environment interaction with deadly consequences. This aligns with the interplay between mutant cancer predisposition genes and asbestos, particularly low exposures, which have been documented in mesothelioma patients [27,28].

## Figures and Tables

**Table 1:** Spontaneous mesotheliomas observed in *Bap1* heterozygous mice and wild-type (WT) littermates enrolled in the lifetime aging study.

Mice with Various *Bap1* Heterozygous Mutations in Different Genetic Backgrounds	Total No. Mice	No. Mice with Mesothelioma	% Mice with Mesothelioma
*Bap1^+/−^*, *Bap1^+/L^*, *Bap1^+/W^* FVB/N [5]	93	2	2.15
*Bap1^+/−^* FVB/N	54	0	0.00
*Bap1^+/−^* C57BL/6	62	0	0.00
*Bap1^+/−^* 129/Sv	59	0	0.00
*Bap1^+/−^* C57BL/6 (Genentech)	61	0	0.00
Total Bap1-mutant mice	329	2	0.61
***Bap1^+/+^* (WT) littermates of mice listed above**			
*Bap1^+/+^* FVB/N [5]	43	0	0.00
*Bap1^+/+^* FVB/N	20	0	0.00
*Bap1^+/+^* C57BL/6	57	0	0.00
*Bap1^+/+^* 129/Sv	43	0	0.00
*Bap1^+/+^* C57BL/6 (Genentech)	64	0	0.00
Total WT littermates	227	0	0.00

**Table 2:** Historical control dataset of wild-type FVB/N mice followed for a lifetime.

Study	Total No. Mice	No. Mice with Mesothelioma	% Mice with Mesothelioma	Follow-Up Time
Huang et al., 2008 [20]	234	0	0.00	128.5 weeks
Panchenko et al., 2016 [21]	69	0	0.00	Lifetime (−132 weeks)

## Data Availability

All data supporting the reported results are found in the report.

## Abbreviations

AIC	Akaike Information Criterion
*Bap1*	Mouse BRCA1-Associated Protein-1 Gene
*BAP1*	Human BRCA1-Associated Protein-1 Gene
BAP1-TPDS	BAP1 Tumor Predisposition Syndrome
CI	Confidence Interval
GEMMs	Genetically Engineered Mouse Models
HCD	Historical Control Dataset
IACUC	Institutional Animal Care and Use Committee
IHC	Immunohistochemical
MAP	Meta-Analytic Predictive
MCMC	Markov Chain Monte Carlo
ML	Maximum-Likelihood
MPM	Malignant Pleural Mesothelioma
OR	Odds Ratio
PV	Pathogenic Variant
RD	Risk Difference
RR	Risk Ratio
WT	Wild-Type
ZFN	Zinc Finger Nuclease
